# Ischemic Penumbra Protection: Beyond Brains or Not

**DOI:** 10.1002/mco2.70370

**Published:** 2025-09-09

**Authors:** Cesario V. Borlongan, Kaya Xu

**Affiliations:** ^1^ Department of Neurosurgery The Affiliated Hospital of Guizhou Medical University Guiyang Guizhou China; ^2^ Stem Cell Biomedical, Inc. Wildwood USA; ^3^ Center of Excellence for Aging and Brain Repair, Department of Neurosurgery and Brain Repair University of South Florida College of Medicine Tampa Florida USA; ^4^ Department of Hyperbaric Oxygen The Affiliated Hospital of Guizhou Medical University Guiyang Guizhou China

1

In a recent study published in MedComm, Chuanjie Wu et al. [1] reported important roles of transmembrane protein 30A (Tmem30a) and annexin V after ischemic stroke, in which treatment with recombinant annexin V reduced apoptosis in the penumbra and improved neurologic outcomes in a Tmem30a‐dependent way. This study highlighted both the importance of communication between the central nervous system (CNS) and body organs after ischemic stroke and the necessity of the existence of penumbra in the communication.

Brain diseases represent a considerable social and economic burden worldwide. Patients suffering from brain diseases, including but not limited to stroke, traumatic brain injury (TBI), Alzheimer's disease (AD), Parkinson's disease (PD), are still increasing despite great research advancements and emerging novel therapies. Previously, the CNS was regarded as a separate compartment, which was isolated from the rest of the body. Exogenous pharmacological modalities were unable to cross the blood–brain barrier (BBB). However, accumulating evidence over the past few years indicates that the CNS bidirectionally interacts with other major organs [2]. These axes that mediate communications between the brain and body organs play an important role in maintaining homeostasis after brain diseases (Figure 1A). These axes also hold great potential for the exploration of novel mechanisms and therapeutic targets for brain diseases. The mediators might serve as new targets of brain protection.

**FIGURE 1:**
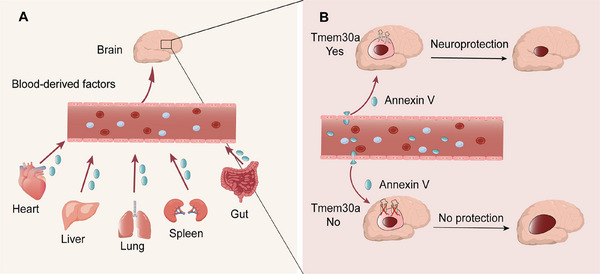
Brain tissue per se mounts in the crosstalks after brain diseases. (A) Brain communicates with major organs through several axes after brain diseases. Blood‐borne factors serve as mediators for the inter‐organ communications. (B) The beneficial effects of blood‐borne factor, annexin V (in blue), in the plasma, are highly dependent on the existence of Tmem30a expression in the penumbra (in light pink) after stroke onset. Annexin V (in blue) in the plasma prevents the increase of the infarct (in brown) when there is Tmem30a in the penumbra. In contrast, annexin V in the plasma could not prevent the increase of the infarct (in brown) when there is no Tmem30a in the penumbra. Dependent on the existence of Tmem30a in the penumbra, annexin V exerts beneficial effects after ischemic stroke.

CNS–body organ communications have been gradually recognized and accepted in recent years due to sensitive proteomics and single‐cell RNA sequencing technologies, as well as the development of new experimental disease model systems. Several key types of mediators, including a broad array of signaling cytokines, nucleic acids, lipids, microbiomes, and metabolites, serve as the bridge for crosstalks between the brain and body organs. Increasing evidence has shown that these mediators exert a beneficial effect in many brain diseases. For example, plasma collected from voluntarily running mice could reduce baseline neuroinflammatory gene expression when infused into sedentary mice [3]. Additionally, the complement cascade inhibitor, clusterin, could bind to brain endothelial cells and reduce neuroinflammatory gene expression in a mouse model. More importantly, patients with cognitive impairment displayed a higher level of plasma clusterin after a 6‐month exercise. But not all patients exhibited improvement in cognitive function. These findings support the idea that the CNS–body crosstalk might exist, but the cytoprotective effects were variable among patients. Thus, these beneficial effects might also be closely associated with the micro‐environment in the brain.

In the past 20 years, clinical neuroimaging tools have been able to discriminate the penumbra tissue with reduced blood flow but preserved metabolism, which differs from that of infarct tissue. The penumbra theory serves as the basis of recanalization therapy for ischemic stroke. However, only half of ischemic stroke patients benefited from the recanalization therapy. This study further implicated two potential but not mutually exclusive conditions of penumbra. The penumbra is a mixture of different nerve cells in various states after ischemic onset. Recent evidence indicates that differential vulnerability exists among cell types in the penumbra after stroke onset. Neurons are most vulnerable to hypoxia, followed by endothelial cells and astrocytes [4]. All these cell types activate genes relating to autophagy, apoptosis, and necroptosis, but the specific genes vary across these various cell types in the penumbra [4]. These data indicate that careful consideration should be given to the penumbra tissue, in which differential vulnerability and response to treatments may affect cell fate and function outcomes in stroke patients.

Equally impactful in this study was the discovery that annexin V, as a blood‐borne factor, served as a mediator for neuroprotection after ischemic stroke [5]. Annexin V is a widely distributed class of calcium‐dependent phospholipid‐binding protein. Previous studies have revealed that annexin V exists in human and mouse plasma, but generally at a low concentration [5]. However, myocardial ischemia led to a transient and significant increase in annexin V in patients’ plasma. Moreover, a higher level of annexin V was strongly associated with a good clinical prognosis in patients with myocardial ischemia or ischemic stroke. Additionally, annexin V had the advantage of crossing the BBB because of its small molecular weight. Altogether, these studies indicated that annexin V may be a new target for neuroprotection after ischemic stroke.

Future studies are warranted to identify additional crucial mediators from major organs outside the brain after ischemic stroke, especially those that have specific receptors or targets in the brain. Similarly, cell type‐specific markers, which are closely associated with various cell fates in the penumbra after stroke onset, are also needed to discriminate the functional outcome of the penumbra. For example, multiple molecules have been discovered in the ischemic penumbral region because of the wide application of omics analysis, especially the rapid development of spatial transcriptomics and proteomics. Thus, the strategy of interventions in the peripheral systems will bring more benefits to the brain in the near future when further considering the functional status of the brain.

## Author Contributions

C.B. drafted the manuscript and drew the figure. K.X. reviewed the manuscript. Both authors read and approved the final version of the manuscript.

## Ethics Statement

The authors have nothing to report.

## Conflicts of Interest

Author Cesario V Borlonga is an employee in Stem Cell Biomedical, Inc., but has no potential relevant financial or non‐financial interests to disclose. The other authors have no conflicts of interest to declare.

## Data Availability

The authors have nothing to report.
